# Puffy Hand Syndrome Revealed by a Severe Staphylococcal Skin Infection

**DOI:** 10.1155/2013/376060

**Published:** 2013-10-02

**Authors:** Reyhan Amode, Paul Bilan, Carole Sin, Anaïs Marchal, Michèle-Léa Sigal, Emmanuel Mahé

**Affiliations:** Service de Dermatologie, Hôpital Victor Dupouy, 69 rue du Lieutenant-Colonel Prud'hon, 95100 Argenteuil, France

## Abstract

Puffy hand syndrome develops after long-term intravenous drug addiction. It is characterized by a nonpitting edema, affecting the dorsal side of fingers and hands with puffy aspect. Frequency and severity of the complications of this syndrome are rarely reported. Local infectious complications such as cellulitis can be severe and can enable the diagnosis. Herein, we report the case of a 41-year-old man who went to the emergency department for abdominal pain, fever, and bullous lesions of legs and arms with edema. Bacteriologic examination of a closed bullous lesion evidenced a methicillin sensitive *Staphylococcus aureus*. The abdomen computed tomography excluded deep infections and peritoneal effusion. The patient was successfully treated by intravenous oxacillin and clindamycin. He had a previous history of intravenous heroin addiction. We retained the diagnosis of puffy hand syndrome revealed by a severe staphylococcal infection with toxic involvement mimicking a four limbs cellulitis. Puffy hand syndrome, apart from the chronic lymphedema treatment, has no specific medication available. Prophylactic measures against skin infections are essential.

## 1. Introduction

Puffy hand syndrome develops in long-term intravenous drug users. Frequency and severity of the complications of this syndrome are rarely reported. We report here a case of puffy hand syndrome revealed by a severe staphylococcal infection with toxic complications mimicking a four limbs cellulitis.

## 2. Case Presentation

A 41-year-old man was admitted to our institution for bilateral feet, legs, arms, and hand edema with fever. He had a previous history of HCV hepatitis and intravenous heroin addiction cured fifteen years ago. Heroin addiction was substituted by buprenorphine, without buprenorphine intravenous injection. 

He had been reporting progressive feet and hands edema for several years, which became permanent for six months. He saw his general practitioner for worsening of edema with erythema. He was prescribed paracetamol and a nonsteroidal anti-inflammatory drug. Five days later, he went to the emergency department for abdominal pain and bullous lesions of legs and arms. He had a 39°C fever and severe sepsis clinical criteria. 

**Figure 1:**
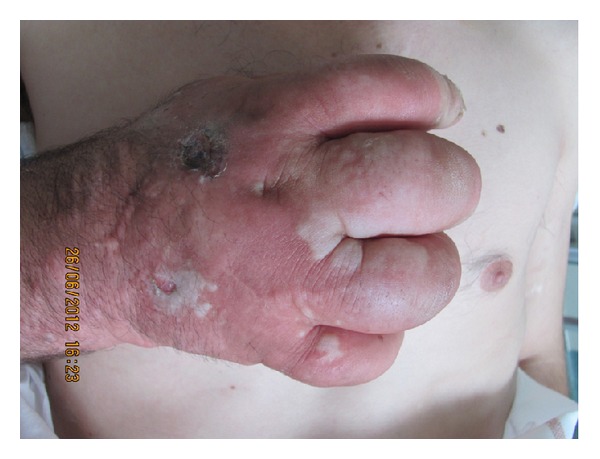
Puffy hand syndrome with large bullous lesions and erythema.

He had nonpitting edema with erythema of feet, legs, hands, and forearms. Several bullous lesions affected his hands and feet (Figure 1) without formal argument for a necrotizing fasciitis or a staphylococcal scalded skin syndrome.

Blood cells count revealed hyperleukocytosis, C reactive protein was increased, and the patient suffered from acute renal failure. Bacteriologic examination of a closed bullous lesion evidenced a methicillin sensitive *Staphylococcus aureus*. HIV, B hepatitis, and syphilis serologies were negative. The abdomen computed tomography excluded deep abscesses or peritoneal effusion. 

The patient was successfully treated by hemodynamic support and intravenous oxacillin and clindamycin. Erythema and abdominal pain regressed within a few days. Two months later, we confirmed persistent feet and hands edema. Lower and upper limb venous doppler excluded deep venous thrombosis. So, we concluded it as a puffy hand syndrome revealed by a severe staphylococcal infection with toxic involvement. The nonsteroidal anti-inflammatory treatment could have been an aggravating factor. The four limbs simultaneous erythema and bullous lesions, the abdominal pain, and the severe sepsis were considered to be toxic complications.

## 3. Discussion

Puffy hand syndrome is a long-term complication of intravenous drug abuse. Firstly described by Abeles in 1965 in New York prisoners, it could affect from 7 to 16% intravenous drug users [1, 2]. Sex (women), injections in the hands and in the feet and the absence of tourniquet are significant risk factors for puffy hand syndrome [3]. It starts during or after a long period of intravenous drug addiction with intermittent painless edema. After several months of evolution, the edema becomes permanent and does not decrease with the elevation of the upper limb. It is a nonpitting or slightly pitting, affecting the dorsal side of fingers and hands with puffy aspect, sometimes asymmetric (more important in the nondominant member), with skin thickness. Feet involvement is less frequent, related to the lower limbs injections [4]. Acrocyanosis and Raynaud phenomenon have been reported [5]. Local infectious complications such as cellulitis can be severe and frequently enable the diagnosis. Indeed, the patients suffering from puffy hand syndrome rarely consult for the edema due to the guilt feelings and despite the functional, aesthetic, and social disagreements. 

The physiopathology of puffy hand syndrome is multifactorial. It seems to involve venous insufficiency and lymphatic insufficiency. Venous injuries caused by the injections result in repeated superficial venous thromboses until the destruction of the whole superficial venous network [6, 7]. Numerous adjuvants such as quinine, crushed glass, or plaster are frequently used. Quinine is well known to be toxic against veins [8] and probalby against lymphatic vessels [9]. Buprenorphine intravenous misuse has also been suspected to be involved in lymphatic destructions due to its lack of solubility, but this hypothesis has not been statistically confirmed [3]. Furthermore, it has been demonstrated by lymphoscintigraphy evaluation that skin infections, common in drug addicts, provoke lymphatic damages; most patients with repeated erysipelas have significant and even permanent abnormalities in regional lymphatic drainage [10].

## 4. Conclusion

Puffy hand syndrome has no specific treatment available. Chronic lymphedema treatment is based on low-stretch bandaging and wearing elastic garment [11]. Prophylactic measures against skin infections are essential.
